# Chicken Heads as a Promising By-Product for Preparation of Food Gelatins

**DOI:** 10.3390/molecules25030494

**Published:** 2020-01-23

**Authors:** Robert Gál, Pavel Mokrejš, Petr Mrázek, Jana Pavlačková, Dagmar Janáčová, Jana Orsavová

**Affiliations:** 1 Department of Food Technology, Faculty of Technology, Tomas Bata University in Zlín, Vavrečkova 275, 760 01 Zlín, Czech Republic; gal@utb.cz; 2 Department of Polymer Engineering, Faculty of Technology, Tomas Bata University in Zlín, Vavrečkova 275, 760 01 Zlín, Czech Republic; p_mrazek@utb.cz; 3 Department of Lipids, Detergents and Cosmetics Technology, Faculty of Technology, Tomas Bata University in Zlín, Vavrečkova 275, 760 01 Zlín, Czech Republic; pavlackova@utb.cz; 4 Department of Processing Control and Applied Computer Science, Faculty of Applied Informatics, Tomas Bata University in Zlín, Nad Stráněmi 4511, 760 05 Zlín, Czech Republic; janacova@utb.cz; 5 Linguae Centre, Faculty of Humanities, Tomas Bata University in Zlín, Štefánikova 5670, 760 01 Zlín, Czech Republic; orsavova@utb.cz

**Keywords:** by-products, chicken heads, enzyme technology, extraction, food applications, gelatin, innovative process, sustainability

## Abstract

Every year, the poultry industry produces a large number of by-products such as chicken heads containing a considerable proportion of proteins, particularly collagen. To prepare gelatin is one of the possibilities to advantageously utilize these by-products as raw materials. The aim of the paper was to process chicken heads into gelatins. An innovative method for conditioning starting raw material was using the proteolytic enzyme. Three technological factors influencing the yield and properties of extracted gelatins were monitored including the amount of enzyme used in the conditioning of the raw material (0.4% and 1.6%), the time of the conditioning (18 and 48 h), and the first gelatin extraction time (1 and 4 h). The gelatin yield was between 20% and 36%. The gelatin gel strength ranged from 113 to 355 Bloom. The viscosity of the gelatin solution was determined between 1.4 and 9.5 mPa.s. The content of inorganic solids varied from 2.3% to 3.9% and the melting point of the gelatin gel was recorded between 34.5 and 42.2 °C. This study has shown that gelatin obtained from chicken heads has a promising potential with diverse possible applications in the food industry, pharmacy, and cosmetics.

## 1. Introduction

Gelatin is a partial hydrolysate of collagen, which is a high molecular weight polypeptide. It is a water soluble, easily digestible protein composed of 18 amino acids containing all essential amino acids except for tryptophan [1]. Gelatin is a common raw material in the food industry due to its beneficial characteristics such as gel formation, thickening, clarifying, and stabilizing properties [2,3]. It is formed by denaturation of collagen at temperatures above 60 °C, which causes structural changes, intra-molecular bond breaks, and consequent physical-chemical changes. Traditional raw materials in the gelatin production are pork skins and bovine hides since they are universally available [4]. However, as the research shows, gelatin can also be prepared from alternative sources, such as poultry [5,6,7,8], fish [9,10,11], goat meat [12,13], or even insects [14]. Chosen method for the production defines the type of gained gelatin. Type A is obtained by acid conditioning and type B by alkaline. Gelatin quality is assessed by the strength of gelatin gel (expressed in Bloom values) and viscosity of gelatin solution [15]. According to Bloom value, gelatin is classified as low (below 150 Bloom), medium (from 150 to 220 Bloom), or high (from 220 to 300 Bloom) gel strength gelatin [16]. If gelatin does not form a gel, it is a collagen hydrolysate with possible applications, e.g., in food industry, such as a nutritional supplement, a thickening agent, or a binder.

A great emphasis is placed on the maximum promotion of waste reduction and utilization of raw materials in various industries. This is particularly true for the meat-processing industry, which produces large amounts of by-products such as blood, bones, meat cuttings, skins, adipose tissues, horns, hooves, feet, skulls, and intestines [17]. Regarding poultry meat, carcasses are processed in several consecutive operations. The first procedure involves hanging up, stunning, bleeding, steaming, plucking, and refining. This is followed by gutting, cutting the neck skin under the head and along the neck, removing the head, crop, esophagus, and trachea out of the thoracic cavity, cutting the feet and abdominal cavity, cleaning the stomach, etc. Cooling follows gutting immediately. Carcasses and offal are inspected by veterinary inspectors. Distribution and packaging ensure grading according to weight and quality [18].

Companies operating slaughterhouses must deal with livestock by-products not intended for human consumption by methods defined by Regulation (EC) No 1069/2009 [19]. This prescribed disposal of by-products increases costs of the whole production [20]. However, these waste by-products have a significant potential since they are rich in proteins, especially collagen, which can be processed into collagen products with further industrial applications. By-products may also be used as raw materials in the production of biomolecules, such as protein hydrolysates with bioactive properties [21]. Such hydrolysates are suitable alternatives to soy proteins because they do not contain anti-nutritional factors or allergenic proteins [22]. Approximately 25 million tons of by-products in the United States and 15 million tons in the European Union are annually used in the production of valuable fats and proteins [23].

The poultry-processing industry is one of the fastest growing food sectors with an annual worldwide production of 120 million tons of chicken [24]. Poultry by-products are either liquid or solid, edible, or inedible and account for up to 30% of the total production. Liquid by-products include blood, which can be processed into meal or utilized as an ingredient in the meat production. It is also a rich source of large amounts of proteins, especially hemoglobin [25]. Blood in adult poultry comprises 7.5% of the total weight and contains approximately 85% water. The rest are proteins, lipids, and minerals. It is obtained during the bleeding procedure and is very valuable from a nutritional point of view. To avoid clotting after bleeding, it is essential to stabilize or defibrillate it [26]. It is mostly used in the livestock feed and pharmaceutical industry, and less in the food industry. Skin, forms 7–10% of the total weight, and is a typical representative of a solid by-product used generally in the gelatin production or as a part of meat products. It is a protective barrier against undesirable environmental effects. Fat deposited under the skin must be removed due to specific dietary requirements for poultry [27]. Hearts, livers, and stomachs are further by-products with various possible culinary purposes. Intestines and glands may be employed in the production of hormones and enzymes. Chicken feet are a source of gelatin and fat. Inedible by-products, which cannot be directly applied in human consumption, include bones that may be processed into meal or feathers used in the fertilizers production or bedding. Other waste by-products and derivatives are generally utilized in the production of livestock feed and fertilizers. Poultry heads, which are also by-products not intended for direct human consumption, account for approximately 2% of the total weight and are used in the livestock feed production or processed into meal. Since they can become a potential raw material in the gelatin production, their value for the poultry industry may rise. However, only a few researchers have been examining methods to prepare gelatin from poultry heads. The preparation of gelatin from chicken and turkey heads and their different functional properties was described [28].

From increasingly stringent legislative measures and changing attitudes of state authorities and consumers alike, increasing pressures on gelatin producers in the use of biotechnological methods of gelatin production, which will be safely and environmentally-produced using these innovative biotechnological methods, can be expected. Nevertheless, the biotechnological production process of gelatins is not yet a common commercial matter. Therefore, the presented paper focusing on processing chicken by-products by biotechnological methods into gelatins may contribute to more efficient sustainability in the food industry. The main purpose of the work is to process chicken heads into gelatins using an innovative method for conditioning of purified collagen by the action of a specific proteolytic enzyme. Furthermore, the purpose of this work is to detail and examine some technological conditions during gelatin processing on the properties of prepared gelatins. The specific hypothesis being tested is that chicken heads can be processed into high-bloom value gelatins with optimal efficiency of the process (gelatin yield).

## 2. Results and Discussion

A schedule of experiments and results of the processing of chicken heads into gelatins are given in Table 1.

**Figure 1:**
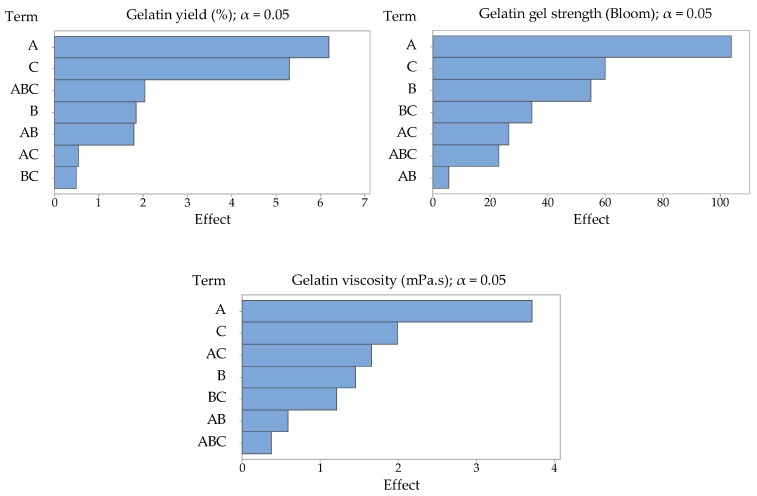
Pareto charts of the effects of studied process factors (A–amount of enzyme, B–enzyme conditioning time, and C–extraction time) and their interactions on gelatin yield, gelatin gel strength, and gelatin viscosity.

Statistical significance of the studied process factors in the observed limits was evaluated using the standard Fisher’s significance test and the P-values for a 95% confidence level. For the factor schemes used by us, the critical value is F = 10.13, so the higher the F-value is above the critical value, the greater the influence of the process factor is. Similarly, results for *p*-values were evaluated. Factors with a value lower than α = 0.05 have an effect on the evaluated variables with 95% probability. The lower the *p*-value, the greater the influence of the process factor [29]. Pareto charts of the effects of studied process factors—amount of enzyme (factor A), enzyme conditioning time (factor B), extraction time (factor C), and their interactions—on evaluated variables (gelatin yield, gelatin gel strength, and gelatin viscosity) are presented in Figure 1.

### 2.1. Yield of the First Gelatin Fraction

**Figure 2:**
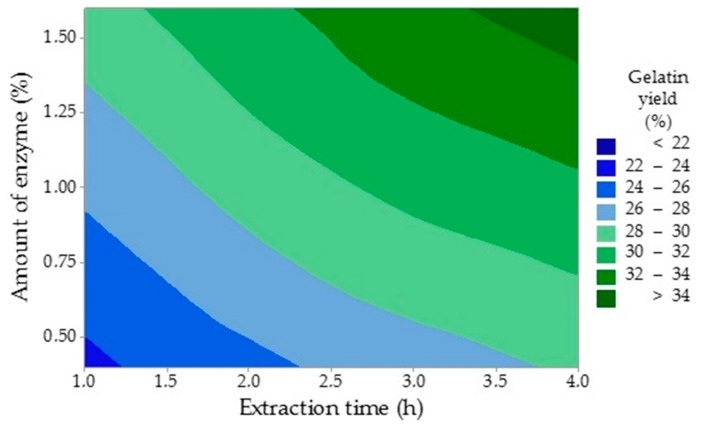
The influence of the time of extraction and the amount of enzyme on the yield of the first gelatin fraction.

The experiment has proved that the time of enzyme conditioning has no significant effect (*p* = 0.209) on the yield of gelatin (Y_G1_). The yield of gelatin in the comparative experiment (19.7%) was 10% lower than in the central experiment (29.7%), which suggests that it is possible to gain a significant amount of gelatin from this raw material even without the enzyme treatment. The amount of enzyme (factor A, *p* = 0.005) and the time of extraction (factor C, *p* = 0.009) have shown the most considerable influence on the extraction efficiency and were statistically significant (*p* ˂ 0.05). The regression equation is Y_G1_ = 17.44 + 5.17 A + 0.0617 B + 1.767 C. Figure 2 shows that longer extraction times and larger amounts of enzyme have increased the yield of gelatin. The yield of the first gelatin fraction ranged from 19.7% to 35.8% and grew with a longer extraction time. Taufik et al. examined the effect of the extraction temperature on properties of chicken feet skin gelatin with the yield varying between 15.3% and 16.5% [30]. It is lower than in this study, which may be due to a lower extraction temperature (45 to 55 °C). Cho et al. investigated the influence of the processing conditions on the properties of gelatins prepared from stingray skin (*Raja Kenojei*) and recorded a similar trend with the yield being between 11.9% and 16.8%, which is comparable with Traufik’s study [31]. The extraction temperature was between 40 and 70 °C. Du et al. studied the properties of gelatin extracted from chicken and turkey heads with a 31.2% yield of the first gelatin fraction (turkey heads) and 24.8% yield (chicken heads), which is in agreement with this study [28]. However, different technological conditions were applied. Raw material was defatted with 0.015 mol/L NaHCO_3_ and pre-treated with 0.1 mol/L NaOH and 0.05 mol/L CH_3_COOH. Thus, this combines alkaline and acidic processes. Gelatin was extracted in two stages at two extraction temperatures: at 50 °C for 18 h and 60 °C for 6 h. Sarbon et al., who investigated gelatin prepared from chicken skin, reported the yield was 16.1%, which is less than in the present study [32]. The extraction temperature was 45 °C and, therefore, could be one of the reasons for such a low gelatin yield. Other authors published the yield of chicken feet gelatin of only 12.6%, which could be the result of the extraction time effect, which was 90 min in their study [33]. Variable values of recorded gelatin yields may be caused by different structures of tested tissues and by variable technological conditions of the gelatin preparation processes especially the extraction temperature and time.

### 2.2. Gelatin Gel Strength

**Figure 3:**
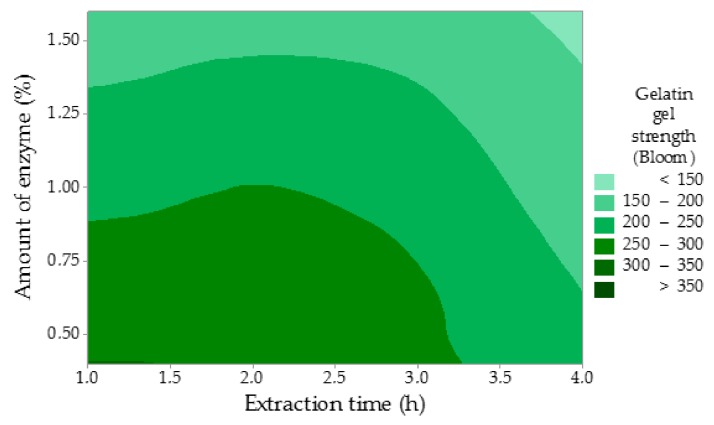
The influence of the time of extraction and the amount of the enzyme on the gelatin strength.

The gelatin gel strength (GS) has been largely influenced by the amount of the enzyme, while the extraction time has been proven to be less significant (Figure 3). In contrast to such a considerable influence of the amount of enzyme (*p* = 0.009, statistically significant, *p* ˂ 0.05), the effect of the time of enzyme conditioning (*p* = 0.079) and the time of extraction (*p* = 0.061) were insignificant. The regression equation is: GS = 407.7 − 86.7 A − 1.833 B − 20.00 C. Nevertheless, the gel strength decreased with rising values of all studied factors. These results suggest that the reduction in the gel strength likely stems from the disruption of the primary structure (peptide bonds) in collagen molecules during the enzyme conditioning of the raw material and the gelatin extraction as well. This disintegration results in a reduction in molecular weight, which causes weakening of the gel network. Higher amounts of the enzyme may also contribute to this process. The highest gel strength of 355 Bloom was measured in Experiment 1, which is 109 Bloom more than in the comparative experiment. The lowest gel strength of 113 Bloom was recorded in Experiment 8 with applied maximum factor limits. Within the rest of the experiments, the gel strength varied between 135 and 248 Bloom, which represents medium or high Bloom gelatins. Du et al. reported the strength of turkey gelatin gel of 369 Bloom, which is a comparable result with Experiment 1 (355 Bloom) in this study, and the strength of chicken gelatin gel of 248 Bloom, which is in accordance with Experiment 3 [28]. Sarbon et al. stated the strength of chicken skin gelatin gel of 355 Bloom when compared to Experiment 1 [32]. Sompie and Triasih published the strength of chicken leg skin gelatin gel with the value of 78 Bloom, which is significantly less than in this study [34]. The reason of such a low gel strength may be a longer extraction time (5 h) if compared to this study (1–4 h). The other also reported lower values of the gelatin gel strength of 79 and 185 Bloom depending on the specific extraction conditions [33].

### 2.3. Gelatin Viscosity

**Figure 4:**
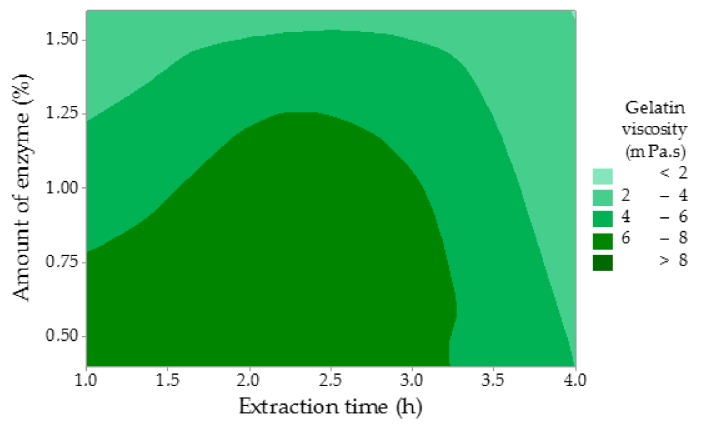
The influence of the time of extraction and the amount of the enzyme on the gelatin gel viscosity.

The highest viscosity (ν) of 9.45 mPa.s was measured in Experiment 1. It suggests that these gelatin properties, both gel strength and viscosity, are closely related. As can be seen in Table 1, viscosity decreased with growing values of all studied factors. However, none of the factors were statistically significant (*p* ˃ 0.05, *p* = 0.055 for the amount of enzyme, *p* = 0.309 for enzyme conditioning time, and *p* = 0.182 for extraction time). The regression equation is: ν = 10.62 − 3.09 A − 0.0485 B − 0.663 C. A similar trend has been observed for the gelatin gel strength. Gelatin with higher viscosity contains longer collagen chains, which causes a rise in the flow resistance if compared to gelatin with a lower viscosity. The lowest viscosity of 1.41 mPa.s was recorded in Experiment 7. Figure 4 displays that higher amounts of the enzyme and longer times of extraction negatively affected the viscosity of the gelatin solution. Sompie and Triasih determined the gelatin viscosity of chicken leg skin gelatin with the value of 6.52 mPa.s, which is comparable with this study [34]. Rafieinan et al. focused on the optimization of the gelatin extraction from chicken deboner residues and recorded the viscosity of 5.85 mPa.s, which is a similar value in Experiment 3 in this study [35]. Viscosity is influenced by both molecular weight and its distribution of the extracted protein. Taufik et al. reported the viscosity of chicken feet skin gelatin ranging from 6.29 to 7.22 mPa.s, which is comparable to other studies [30].

### 2.4. Other Gelatin Parameters and Second Gelatin Extraction

The content of inorganic solids in the gelatin was established ranging from 2.12% to 3.92%. For the application of gelatin in the food industry, mineral content should reach a maximum of 3.0% [36]. This limit was slightly exceeded only in three experiments. A possible reduction of the content of inorganic solids may be achieved by using ion exchangers. Du et al. reached extremely low contents of inorganic solids (0.03% in chicken and 0.06% in turkey heads) [28]. Sarbon et al. also reported a significantly smaller content of 0.4%, which may be due to a lower concentration of NaOH used in the conditioning process [32]. Prepared gelatin solutions had a pH between 7.02 and 7.17, which meets the pH standards for commercial gelatins (3.8 to 7.6) [37,38]. Other studies recorded slightly lower pH between 6.13 and 6.49, which may be caused by the acidic pre-treatment of the raw material in the gelatin preparation [33]. Therefore, studied technological conditions have shown almost no effect on this parameter. The gelatin melting point has been influenced by the presence of the enzyme. Generally, it was higher for a gelatin gel sample with a lower enzyme content (0.4%) and was between 38.8 and 42.2 °C. On the other hand, if the content of the enzyme was 1.6%, the melting point was between 34.9 and 38.6 °C, which is lower than with a smaller content of the enzyme. This may indicate that a higher amount of enzyme causes more extensive disruption of the collagen molecule, which results in melting gelatin at lower temperatures.

The second gelatin extraction provided relatively good yields ranging from 5.55% to 10.6%. However, the quality of this gelatin was lower than the quality of the first gelatin extraction with lower gel strength (<150 Bloom) and viscosity (<2.85 mPa.s).

### 2.5. Design of Optimal Technological Conditions for the Preparation of Gelatin from Chicken Heads

To propose optimal technological conditions, it is necessary to assess the influences of all factors on the efficiency of the preparation and strength of gelatin gel. The aim was to prepare gelatins with a high gel strength (≥220 Bloom) and lower gel strength (≤150 Bloom). The time of enzyme conditioning (24 h) and the amount of the enzyme (0.8%) were set as constant factors. The time of the first extraction was 45 and 120 min. The temperature was 80 °C. The time of the second extraction was 1 and 4 h and the temperature was 95 °C. The most significant parameters of the gelatin quality were determined, such as the gelatin gel strength and the viscosity. Table 2 shows the results of these optimization experiments. They have confirmed the importance of the extraction time for the quality of the prepared gelatin. The strength of the gelatin gel prepared from gelatin extracted for 45 min was 277 Bloom, whereas it was only 140 Bloom within the extraction time of 120 min. The extraction yield was 7% lower in a 45-min extraction compared to a 120-min extraction. The yields from the second extraction were higher than 10%, which confirmed the effect of high temperature on the yield of gelatin.

## 3. Materials and Methods

### 3.1. Appliances, Tools, and Chemicals

Raciola Ltd. (Uherský Brod, Czech Republic) supplied chicken heads. First, raw material analyses were performed determining dry matter content (23.0 ± 0.1%). Then, the analyses were conducted to determine protein content (50.8 ± 0.5%), collagen proportion in the protein content (88.8 ± 1.0%), fat content (33.1 ± 0.4%), and inorganic solids (16.1 ± 0.1%) in dry matter. Each analysis was repeated three times and mean values were calculated.

Stevens LFRA Texture Analyzer for measuring gelatin gel strength (Leonard Farnell and Co Ltd., Liverpool, UK), Ubbelohde viscometer (Technisklo Ltd., Držkov, Czech Republic), meat mincer Braher P22/82 (San Sebastian, Spain), Nedform LT 43 shaker (Valašské Meziříčí, Czech Republic), Kern 440-47 electronic scale, Kern 770 electronic analytical balance (Balingen, Germany), analytical mill IKA A 10 labortechnik (Staufen, Germany), Memmert ULP 400 drying oven (Büchenbach, Germany), Samsung fridge freezer (Seoul, South Korea), Mettler Toledo differential scanning calorimeter DSC 3 (Gießen, Germany), Whatman no. 1 paper (Sigma Aldrich, Gillingham, UK), WTW pH meter Multical pH 526 (Weilheim, Germany), heating board Schott Gerate (Mainz, Germany), and a metal filter sieve with the size of pores of 1 and 2 mm (Labor-komplet, Praha, Czech Republic). Chemicals: NaCl, NaOH, HCl, petroleum ether, acetone, ethanol, and chloroform (Verkon, Praha, Czech Republic). All chemicals were of an analytical grade. The proteolytic enzyme Polarzyme 6.0 T is a serine endoprotease produced by fermentation of microorganisms (Novozymes, Bagsvaerd, Denmark) with an enzyme activity of 6 KPU/g.

### 3.2. Design of Eperiments

The series of experiments were based on the principle of factorial experiments, which can be advantageously used when multiple effects are simultaneously present. It evaluates the effects of one factor and their mutual combinations as well. Another advantage of this method is the reduction of the number of experimental repetitions to the minimum, so that the studied issues can be effectively described without the need to examine all the combinations. The structure of factorial experiments is based on the matrix combining the input values of the experiment. Generally, limit conditions are selected to complement the center conditions. The number of experiments in factorial planning is Np, where N is the number of factor levels and p is the number of factors [39]. This study applied a factorial experiment of 3^2^ type with three factors monitored at two levels. Factor A was the amount of the enzyme (0.4% and 1.6%). Factor B expressed the time of enzyme conditioning of the raw material (18 and 48 h) and Factor C was the time of the first extraction of gelatin (1 and 4 h). The extraction temperature was maintained constant at 80 °C. The central experiment combining mean values of the factors was performed, and supplemented with a comparative experiment under the identical conditions as the central experiment with no amount of enzyme.

Total extraction yield (∑Y) and balance error (BE) were calculated, according to the following formulas.

(1) $$\sum Y=\;Y_{H}+Y_{G1}+Y_{G2}+SR$$

(2) $$BE=\;\left|100-\left(Y_{H}+Y_{G1}+Y_{G2}+SR\right)\right|$$

where *Y*_H_ is the yield of the hydrolysate (%), *Y*_G1_ is the yield of the first gelatin extraction (%), *Y*_G2_ is the yield of the second gelatin extraction (%), and *SR* is a solid residue (%).

Hydrolysate is a liquid fraction after an enzymatic conditioning of collagen starting material. The yield of hydrolysate (*Y*_H_) and yield of two gelatin fractions (*Y*_G1_ and *Y*_G2_) were determined as a percentage of the raw material dry weight. Solid residue is the remaining collagen after the extraction of the second gelatin fraction.

### 3.3. Processing of Chicken Head Collagen Into Gelatins

Preparation consisted of three main technological steps. During the first step, raw material was ground to a particle size of 5 mm using a meat mincer. Prior to further processing, the raw material was frozen at −36 °C and, subsequently, stored at −18 °C. For further processing, it was thawed at 10 °C. Non-collagenous proteins and pigments were removed by mixing the sample with 0.1% NaOH in the ratio of 1:8, which is followed by shaking at room temperature for 45 min. Then it was filtered and rinsed with water. The whole procedure was repeated four times. In the last cycle, distilled water was used instead of NaOH. After filtration and rinsing, raw material was dried at 35 °C for approximately 48 h. After drying, raw material was mixed with a mixture of petroleum ether and ethanol (1:1), which is followed by shaking in four cycles using fresh solvent for each cycle. In the first cycle, the mixture was shaken for 6 h. In the second cycle, the mixture was shaken for 18 h while, in the third cycle, it was shaken for 10 h and for 24 h in the last cycle. Afterward, it was filtered and purified collagen material left to dry in a hood was obtained. The content of dry matter was determined and then it was ground to a particle size of 1–2 mm using an analytical mill.

In the second step, raw material was conditioned by a proteolytic enzyme. Enzymatic conditioning is an eco-friendly alternative to the conventional acid or alkaline conditioning applied in the industry. First, raw material was mixed with distilled water in the ratio of 1:10, which is followed by shaking at room temperature for 15 min. pH was adjusted to 7.5 ± 0.3. Subsequently, proteolytic enzyme Polarzyme 6.0 T was added to the mixture in the amount of 0.4% or 1.6% depending on factor A (Table 1) related to dry matter of raw material. The mixture was shaken for 24 or 72 h, according to factor B at room temperature, with pH being continuously checked and adjusted to maintain the prescribed value if necessary. Then, it was filtered and the content of dry matter in collagen hydrolysate (filtrate) was determined. Collagen material separated on the filter was rinsed thoroughly with water to remove a residual enzyme.

The third step was gelatin extraction. Collagen material was mixed with distilled water in the ratio of 1:10. The mixture was heated to 80 °C and the first gelatin fraction was extracted for 1 or 4 h, according to factor C. The mixture was stirred with a magnetic stirrer continuously during the extraction. Afterward, the mixture was filtered through a G3 filtering crucible and obtained gelatin solution was heated at 100 °C for 5 min to inactivate the remaining enzyme. The extraction of the second gelatin fraction was performed at 95 °C. The samples extracted in the first extraction for 1 h were extracted again for another hour in the second extraction and the samples extracted in the first extraction for 4 h were extracted for a further 15 min. This was followed by drying gelatin solution on a thin layer at 45 °C for approximately 48 h. Film was ground to fine gelatin powder. The insoluble solid residue was dried until its weight remained constant.

### 3.4. Analysis of Gelatin Properties

Prepared gelatins were analyzed for the gel strength, melting point of gelatin gel, inorganic solids, dynamic viscosity, and pH of gelatin solution [40]. To determine the gelatin gel strength (Bloom value), 6.67% of gelatin solution was prepared by mixing 7.5 g of gelatin and 105 mL of distilled water. The mixture swelled for 1–3 h and then was heated to 60 °C in a water bath for 10 min at the longest. Obtained gelatin solution was cooled to room temperature and maintained at 10 °C for 16–18 h. The strength of gelatin gel was measured using a Stevens LFRA texture analyzer. It was expressed by the force required to penetrate the probe at the rate of 1 mm/s into the depth of 4 mm of the surface of the gelatin gel sample. The content of inorganic solids was established by burning the sample in a muffle furnace at 550 °C to the constant weight. The result was expressed by percentage as a proportion of the sample mass after annealing and the sample mass before annealing was multiplied by 100. To determine dynamic viscosity, kinematic viscosity of 6.67% gelatin solution was established first using the Ubbelohde viscometer. The flow time of gelatin solution in a defined capillary section was measured and then the value of dynamic viscosity was calculated using the formula below.

(3) $$\nu =\left(k\cdot \tau -\frac{B}{\tau }\right)\cdot \rho$$

where *ν* is dynamic viscosity (mPa.s), *k* is a viscosity constant detected by calibration fluid (0.5), *τ* is the arithmetic mean of measured flow times (s), *B* is the correction constant for kinetic energy determined from dimensions of the viscometer (2.8), and ρ is gelatin solution density (g/cm^3^).

The density of gelatin solution was 1.003 ± 0.005 g/cm^3^ determined by the pycnometric method. The pH of gelatin solution was determined at the concentration of 1.5% gelatin solution and 35 °C. The melting point of gelatin gel was determined by differential scanning calorimetry (DSC). First, the sample was cooled to 5 °C for 15 min, heated to 50 °C within the rate of 5 °C/min, and then cooled to 5 °C again. The melting point was established from the position of the endothermic peak position of the obtained DSC record.

### 3.5. Statistical Analysis

All analyses were performed in triplicate. Mean and standard deviation values were calculated using Microsoft Office Excel 2013 (Microsoft, Denver, CO, USA). To evaluate obtained data, regression analysis was applied to all results using Minitab^®^ 17.2.1 statistical software for Windows (Fujitsu Ltd., Tokyo, Japan). The level of significance was set to 5% (*p* < 0.05).

## 4. Conclusions

This study investigates the possibilities of the preparation of gelatin from an alternative raw material of chicken heads. It examines the effects of three technological factors: the amount of enzyme during enzyme conditioning (0.4–1.6%), the time of enzyme conditioning (18–48 h), and the time of extraction (1–4 h). The gelatin extraction had two stages. In the first stage, the extraction temperature was set to 80 °C and to 95 °C in the second one. The yield of gelatin in the first extraction was ranged from 20.4% to 35.8%. Higher yields were achieved with higher enzyme levels and longer extraction times. The time of enzyme conditioning showed a less significant effect on the gelatin yield. The values of the gelatin gel strength were recorded between 113 and 355 Bloom and gelatin viscosity ranging from 1.4 to 9.5 mPa.s. These values of the gelatin gel strength and the gelatin solution viscosity decreased with growing amounts of enzyme and longer times of enzyme conditioning and extraction. The melting point of gelatin gels varied from 34.5 to 42.2 °C and was lower if higher enzyme levels were applied during the enzyme conditioning. The content of inorganic solids was recorded between 2.3% and 3.9%. Based on the optimization experiments, two sets of most suitable technological conditions for gelatins preparation were proposed. First, the enzyme amount in the conditioning was set to 0.8%, the time of enzyme conditioning was set to 24 h, and the time of extraction was set to 45 min. Within these conditions, the yield of gelatin was 22.6% and the gel strength was 277 Bloom. In the second experiment design, the amount of enzyme was 0.8%, the time of enzyme conditioning was 24 h, and the time of extraction was 120 min, which resulted in a gelatin yield of 29.9% and a strength of 140 Bloom. Chicken heads perform a significant potential for the preparation of gelatins, which can be applied in the food, pharmaceutical, and cosmetic industry. Solid residues remained after the preparation of gelatins that can be used as well such as in the production of fertilizers. Therefore, chicken heads are very promising raw material considering their economic value.

One of the innovative effects of presented technology is primarily the processing of the starting material by a cheap, commonly available food endoprotease. The use of the enzyme in the conditioning of the starting material is more environmentally-friendly than the use of acids or alkalis applied in the industrial production of gelatins. The presented technology is very convenient for dealing with any animal by-products of slaughter because the constant increase in the consumption of poultry meat leads to an increase in the number of by-products produced. This emphasizes the need for a more efficient sustainability in the food industry (ecological and economical aspects).

## 5. Patents

Related to the work reported in this manuscript following the patent resulted in Patent CZ 307,665–Biotechnology-based production of food gelatin from poultry by-products (1. 2. 2019).

## Figures and Tables

**Table 1:** The experimental design and the results of processing of chicken heads into gelatins.

Exp. No.	Factor A (%)	Factor B (h)	Factor C (h)	Y_H_ (%)	Y_G1_ (%)	Y_G2_ (%)	∑Y (%)	BE (%)	GS (Bloom)	ν (mPa.s)	T_m_ (°C)	IS (%)	pH
1	0.4	18	1	4.12	20.4	7.01	31.53	7.41	355	9.45	41.5	2.33	7.11
2	0.4	18	4	4.21	27.7	6.02	37.93	3.62	211	4.21	38.8	2.84	7.02
3	0.4	48	1	4.43	26.6	7.31	38.34	5.75	248	5.82	39.3	2.45	7.04
4	0.4	48	4	4.31	28.8	8.35	41.46	7.21	219	3.76	42.2	3.53	7.17
5	1.6	18	1	4.85	29.9	6.02	40.77	7.15	207	3.12	38.6	2.76	7.13
6	1.6	18	4	4.88	34.2	6.67	45.75	6.25	162	1.95	34.9	3.31	7.13
7	1.6	48	1	5.26	28.4	7.81	41.47	6.31	135	1.41	37.5	2.98	7.09
8	1.6	48	4	5.15	35.8	8.23	49.18	7.56	113	1.92	35.4	3.92	7.14
9	1	33	2.5	5.12	29.7	5.55	40.37	5.54	245	6.78	34.5	2.35	7.06
10	0	33	2.5	1.71	19.7	10.6	32.01	3.27	246	5.37	38.8	2.12	7.07

Factor A–the amount of enzyme in the conditioning of raw material. Factor B–the time of enzyme conditioning. Factor C–the time of the first extraction of gelatin. Y_H_–the yield of hydrolysate. Y_G1_–the yield of the first gelatin fraction. Y_G2_–the yield of the second gelatin fraction. ∑Y–total extraction yield. BE–balance error. GS–gelatin gel strength. ν–gelatin viscosity. T_m_–gel melting temperature. IS–inorganic solids.

**Table 2:** Design of the optimization experiments and results of characterization of the process, the gel strength, and the viscosity of prepared gelatins.

Exp. No.	Factor A (%)	Factor B (h)	Factor C (min)	Y_H_ (%)	Y_G1_ (%)	Y_G2_ (%)	∑Y (%)	GS (Bloom)	ν (mPa.s)
11	0.8	24	45	4.91	22.6	12.3	39.8	277	5.51
12	0.8	24	120	4.61	29.9	14.1	41.6	140	2.61

Factor A–the amount of enzyme in the conditioning of the raw material. Factor B–the time of the enzyme conditioning. Factor C–the time of the first extraction of gelatin. Y_H_–the yield of hydrolysate. Y_G1_–the yield of the first gelatin fraction. Y_G2_–the yield of the second gelatin fraction. ∑Y–total extraction yield. GS–gelatin gel strength. ν–gelatin viscosity.

## References

[1] Schrieber R., Gareis H. (2007). Gelatine Handbook - Theory and Industrial Practice.

[2] Djagny K.B., Wang Z., Xu S. (2001). Gelatin: A valuable protein for food and pharmaceutical industries, review. Crit. Rev. Food Sci. Nutr..

[3] Gómez-Guillén M.C., Giménez B., López-Caballero M.E., Montero M.P. (2011). Functional and bioactive properties of collagen and gelatin from alternative sources: A review. Food Hydrocoll..

[4] Kittiphattanabawon P., Benjakul S., Visessanguan W., Shahidi F. (2010). Comparative study on characteristics of gelatin from the skins of brownbanded bamboo shark and blacktip shark as affected by extraction conditions. Food Hydrocoll..

[5] Bichukale A.D., Koli J.M., Sonavane A.E., Vishwasrao V.V., Pujari K.H., Shingare P.E. (2018). Functional properties of gelatin extracted from poultry skin and bone waste. Int. J. Pure Appl. Biosci..

[6] Abedinia A., Nafchi A. (2017). Extraction and characterization of gelatin from the feet of Peking duck (*Anas platyrhynchos domestica*) as affected by acid, alkaline, and enzyme pretreatment. Int. J. Biol. Macromol..

[7] Suderman N., Isa M.I.N., Sarbon N.M. (2018). Characterization on the Mechanical and Physical Properties of Chicken Skin Gelatin Films in Comparison to Mammalian Gelatin Films. IOP Conference Series: Materials Science and Engineering.

[8] Erge A., Zorba Ö. (2018). Optimization of gelatin extraction from chicken mechanically deboned meat residue using alkaline pre-treatment. LWT-Food Sci. Technol..

[9] Bower C.K., Avena-Bustillos R.J., Olsen C.W., McHugh T.H., Bechtel P.J. (2006). Characterization of fish-skin gelatin gels and films containing the antimicrobial enzyme lysozyme. J. Food Sci..

[10] Da Trindade Alfaro A., Fonseca G., Prentice C. (2013). 2013: Enhancement of functional properties of wami tilapia (*oreochromis urolepis hornorum*) skin gelatin at different pH values. Food Bioprocess Technol..

[11] Arumugam G.K.S., Sharma D., Balakrishnan R.M., Ponnan Ettiyappan J.B. (2018). Extraction, optimization and characterization of collagen sole fish skin. Sustain. Chem. Pharm..

[12] Mad-Ali S., Benjakul S., Prodpran T., Maqsood S. (2017). Characteristics and gelling properties of gelatin from goat skin as affected by drying methods. J. Food Sci. Technol..

[13] Said M.I., Triatmojo S., Erwanto Y., Fudholi A. (2011). Gelatin properties of goat skin produced by calcium hydroxide as curing material. J. Anim. Sci. Technol..

[14] Mariod A.A., Fadul H. (2015). Preparation and characterization of gelatin from two Sudanese edible insects and its applications in ice cream making. Food Sci. Technol. Int..

[15] Mariod A.A., Adam H.F. (2013). Review: Gelatin, source, extraction and industrial applications. Acta Sci. Pol. Technol. Aliment..

[16] Johnston-Bank F.A. (1983). From tannery to table: An account of gelatin production. J. Soc. Leath. Tech. Chem..

[17] Ryder K., Ha M., Bekhit A.D., Carne A. (2015). Characterisation of novel fungal and bacterial protease preparations and evaluation of their ability to hydrolyse meat myofibrillar and connective tissue proteins. Food Chem..

[18] Owens M.C., Alvorado C., Sams A.R. (2010). Poultry Meat Processing.

[19] Regulation (EC) (2009). No 1069/2009 of the European Parliament and of the Council of 21 October 2009 Laying Down Health Rules as Regards Animal by-Products and Derived Products Not Intended for Human Consumption and Repealing Regulation (EC) No 1774/2002 (Animal By-Products Regulation).

[20] Toldrá F., Aristoy M.-C., Mora L., Reig M. (2012). Innovations in value-addition of edible meat by-products. Meat Sci..

[21] Lasekan A., Abu Bakar F., Hashim D. (2013). Potential of chicken by-products as sources of useful biological resources. Waste Manag..

[22] Martínez-Alvarez O., Chamorro S., Brenes A. (2015). Protein hydrolysates from animal processing by-products as a source of bioactive molecules with interest in animal feeding: A review. Food Res. Int..

[23] Hamilton C.R. Real and Perceived Issues Involving Animal Proteins. Protein Sources for the Animal Feed Industry (FAO Report). http://www.fao.org/tempref/docrep/fao/007/y5019e/y5019e00.pdf.

[24] Poultry Slaughter 2017 Summary. National Agricultural Statistics Service. https://downloads.usda.library.cornell.edu/usda-esmis/files/pg15bd88s/br86b638d/4f16c564f/PoulSlauSu-02-26-2018.pdf.

[25] Ofori J.A., Hsieh Y.H. (2014). Issues related to the use of blood in food and animal feed. Crit. Rev. Food Sci. Nutr..

[26] Jayathilakan K., Sultana K., Radhakrishna K., Bawa A.S. (2012). Utilization of by-products and waste materials from meat, poultry and fish processing industries. J. Food Sci. Technol..

[27] Irshad A., Sharma B. (2015). Abbatoir by-product utilization for sustainable meat industry: A review. J. Animal Prod. Adv..

[28] Du L., Khiari Z., Pietrasik Z., Betti M. (2013). Physicochemical and functional properties of gelatins extracted from turkey and chicken heads. Poult. Sci..

[29] Politis S.N., Colombo P., Colombo G., Rekkas D.M. (2017). Design of experiments (DoE) in pharmaceutical development. Drug Dev. Ind. Pharm..

[30] Taufik M., Triatmojo S., Erwanto Y., Santoso U. (2010). Effect of Broiler Age and Extraction Temperature on Characteristic Chicken Feet Skin Gelatin. Proceedings of the 5th International Seminar on Tropical Animal Production.

[31] Cho S., Jahncke M.L., Chin K.B., Eun J.B. (2006). Effect of processing conditions on the properties of gelatin from skate (*Raja Kenojei*) skins. Food Hydrocoll..

[32] Sarbon N.M., Nazlin F.B., Howell K. (2013). Preparation and characterisation of chicken skin gelatin as an alternative to mammalian gelatin. Food Hydrocoll..

[33] Widyasari R., Rawdkuen S. (2014). Extraction and characterization of gelatin from chicken feet by acid and ultrasound assisted extraction. Food Appl. Biosci. J..

[34] Sompie M., Triasih A. (2017). Effect of extraction temperature on characteristics of chicken legskin gelatin. Proceedings of the IOP Conference Series: Earth and Environmental Science, Proceedings of the International Symposium on Food and Agro-Biodiversity (ISFA).

[35] Rafieian F., Keramat J., Kadivar M. (2011). Optimization of gelatin extraction from chicken deboner residue using RSM method. J. Food Sci. Technol..

[36] Food Chemical Codex (FCC). https://www.foodchemicalscodex.org/.

[37] (2011). United States Pharmacopoeia 35 NF 30.

[38] European Pharmacopoeia 9.0 (2017). European Directorate for the Quality of Medicines & Health Care.

[39] Antony J. (2003). Design of Experiments for Engineers and Scientists.

[40] (2019). Standard Testing Methods for Edible Gelatin. http://www.gelatin-gmia.com.

